# Selenomethionine Alleviates Deoxynivalenol-Induced Oxidative Injury in Porcine Intestinal Epithelial Cells Independent of MAPK Pathway Regulation

**DOI:** 10.3390/antiox13030356

**Published:** 2024-03-16

**Authors:** Zhouyin Huang, Haopeng Zhong, Ting Li, Zirui Wang, Xingping Chen, Tiande Zou, Jinming You, Jun Chen

**Affiliations:** Jiangxi Province Key Laboratory of Animal Nutrition, College of Animal Science and Technology, Jiangxi Agricultural University, Nanchang 330045, China; hzy11373x@stu.jxau.edu.cn (Z.H.); zhonghaopeng@stu.jxau.edu.cn (H.Z.); lt1330082538@stu.jxau.edu.cn (T.L.); wangzirui@jxau.edu.cn (Z.W.); cxp0315@jxau.edu.cn (X.C.); tiandezou@jxau.edu.cn (T.Z.)

**Keywords:** deoxynivalenol, MAPK pathway, oxidative injury, porcine intestinal epithelial cells, selenomethionine

## Abstract

Deoxynivalenol (DON) is a prevalent contaminant in feed and food, posing a serious threat to the health of both humans and animals. The pig stands as an ideal subject for the study of DON due to its recognition as the most susceptible animal to DON. In this study, the IPEC-J2 cells were utilized as an in vitro model to explore the potential of SeMet in alleviating the intestinal toxicity and oxidative injury in intestinal epithelial cells when exposed to DON. Cells were treated either with or without 4.0 μM SeMet, in combination with or without a simultaneous treatment with 0.5 μg/mL DON, for a duration of 24 h. Then, cells or related samples were analyzed for cell proliferation, lactate dehydrogenase (LDH) release, reactive oxygen species (ROS) level, gene expressions, and protein expressions. The results showed that SeMet mitigated the cellular toxicity caused by DON, evidenced by elevated cell proliferation and the reduced LDH release of IPEC-J2 cells in the SeMet + DON group vs. the DON group. Moreover, the SeMet treatment markedly promoted antioxidant functions and decreased the oxidative injury in IPEC-J2 cell, which is indicated by the decreased ROS level and up-regulated mRNA levels of *GPX1*, *TXNRD1*, *Nrf2*, and *GCLC* in IPEC-J2 cells in the SeMet + DON group vs. the DON group. However, in both the absence and presence of exposure to DON, the SeMet treatment did not affect the protein expression of MAPK (JNK, Erk1/2, and P38) and phosphorylated MAPK (p-JNK, p-Erk1/2, and p-P38) in IPEC-J2 cells. Collectively, SeMet alleviated the DON-induced oxidative injury in porcine intestinal epithelial cells independent of the MAPK pathway regulation.

## 1. Introduction

Deoxynivalenol (DON), commonly known as vomitoxin, is a secondary metabolite produced by the *Fusarium* fungi, categorized as a type B trichosporene [1]. DON is extensively found within various crops, food products, and animal feed, exerting a severe impact on the well-being of both humans and livestock [1]. More importantly, it should be noted that DON possesses a formidable stability that is resistant to decomposition by gastric juice [2]. A previous study has shown that DON possesses the most prominent concentration of mycotoxin in animal feed, with levels reaching 4 mg/kg [3]. Furthermore, DON exhibits a diverse range of toxic impacts on both human beings and animals [4]. The susceptibility to DON varies across different species, with the pig being identified as the most vulnerable animal [5]. As a result, pigs are a perfect model for studying the detrimental consequences of DON.

The primary site for the nutrient digestion and absorption is the intestinal tract, which also serves as the initial line of defense against mycotoxin invasion. The intestinal epithelial cells, as an integral component of the intestinal barrier, play a significant role in this process. It is demonstrated that the contamination of DON in animal feed could decrease feed intake and intestinal nutrient absorption [6,7]. Additionally, it could cause intestinal inflammation, imbalances in microbial homeostasis, and oxidative stress in the intestines [6,7]. Hence, the intestinal tract can be regarded as a significant target of DON, along with being a target for nutritional modulation in order to mitigate the toxic impacts of DON [1].

Selenium constitutes one of the indispensable trace elements found in both humans and animals, including pigs. Selenium, serving as a constituent of mammalian enzymes like glutathione peroxidase and other selenoproteins, assumes a crucial role in numerous physiological reactions, such as augmenting immunity, fortifying stress resistance, detoxification, and cancer prevention [8,9,10]. In the realm of pig feed, the supplementation of selenium takes place through the inclusion of both inorganic and organic forms [11]. Inorganic selenium, particularly sodium selenite, constitutes the primary source, while organic selenium is predominantly derived from yeast selenium, with its principal form being selenomethionine (SeMet) [11]. Compared with sodium selenite, SeMet exerts a range of important advantages, including less toxicity and higher bioavailability [12]. SeMet could replace methionine to incorporate into protein synthesis or can be directly converted into selenocysteine through the trans-sulfuration pathway [12]. Indeed, it is reported that SeMet supplementation enhanced the antioxidant capacity, immunity, and selenium deposition in pigs when compared to sodium selenite [13,14,15]. Therefore, SeMet has more advantages over sodium selenite as a selenium source in swine nutrition.

It has been reported that selenium can alleviate the DON-induced oxidative damage of splenic lymphocytes by improving the glutathione peroxidase activity in piglets [16]. According to another study, sodium selenite has been found to have the capability of diminishing the negative impact of DON on GPX1-knockout pig lymphocytes [17]. Most recently, selenium nanoparticles have been reported to effectively mitigate the intestinal epithelial barrier dysfunction induced by DON, through the regulation of endoplasmic reticulum stress in IPEC-J2 cells [18]. Furthermore, it has been found that SeMet ameliorated the oxidative stress elicited by zearalenone through the modulation of the Nrf2/Keap1 signaling pathway in IPEC-J2 cells [19]. However, less is known about the potential of SeMet as a selenium source for mitigating the intestinal toxicity and oxidative damage caused by DON in pigs, considering their high susceptibility to this toxin.

Intracellular signaling pathways, which are triggered in response to excessive oxygen radicals, play pivotal roles in combating oxidative stress [20]. The mitogen-activated protein kinase (MAPK) signaling pathways are involved in diverse physiological processes (such as cell growth, migration, proliferation, inflammation, and apoptosis) [21], and are critical for the induction of cellular oxidative stress responses [20]. More interestingly, the potential mechanism of the DON exposure toxicity has been associated with the selective activation of MAPK pathways and oxidative stress regulation [22,23,24]. Nevertheless, it remains unclear whether SeMet could regulate cellular MAPK pathways to combat the oxidative stress injury induced by DON in IPEC-J2 cells. Therefore, the IPEC-J2 cells were utilized as an in vitro model to test the hypothesis that SeMet could effectively alleviate the intestinal toxicity and oxidative stress injury by potentially regulating the MAPK pathway in intestinal epithelial cells when exposed to DON.

## 2. Materials and Methods

### 2.1. Chemical and Reagents

The DON (with a purity level of at least 98%) and SeMet (with a purity level of at least 99%) were acquired from Sigma Chemical Company (St Louis, MO, USA).

### 2.2. Cell Culture

All animal cell protocols used in this study were approved by the Institutional Animal Care and Use Committee of Jiangxi Agricultural University (Ethical code: JXAULL-20220627). The IPEC-J2 cells utilized in this study was graciously donated by Dr. Xiangfang Zeng from China Agricultural University, Beijing, China. The IEPC-J2 cells were initially isolated from the jejunal epithelia of a neonatal unsuckled piglet by Schierack et al. (2006) [25]. The IPEC-J2 cells were cultured following the methodology outlined in our previous study [26]. Briefly, the cells were cultured in Dubecco’s Modified Eagel Medium/Ham’s F12 (Gibco, Grand Island, NY, USA), which was supplemented with 10% fetal bovine serum (Gibco, Grand Island, NY, USA) and 1% penicillin/streptomycin (Gibco, Grand Island, NY, USA). The cells were kept in a humidified environment with 5% CO_2_ at 37 °C, and the medium was renewed on a bi-daily basis [26]. The IPEC-J2 cells used in this research underwent 60~70 passages before the administration of SeMet and/or DON treatment.

### 2.3. Cell Viability Analysis

The cell viability was measured using the Cell Counting Kit-8 assay (CCK-8) (Beyotime, Shanghai, China). Specifically, cells were seeded into 96-well microplates at a concentration of 1 × 10^4^ cells/well and maintained in a complete medium at 37 °C and 5% CO_2_ until they reached approximately 80% confluence. Subsequently, the cells were treated with various concentrations of DON (0, 0.10, 0.25, 0.50, 1.0, and 2.0 μg/mL) or SeMet (0, 0.5, 1.0, 2.0, 4.0, or 8.0 μM) for 24 h. Alternatively, cells were cotreated with DON (0.50 μg/mL) and various concentrations of SeMet (0, 0.50, 1.0, 2.0, 4.0 or 8.0 μM) for 24 h. Thereafter, the CCK-8 reagent was added and incubated for an additional 1 h. Finally, the absorbance was measured at a wavelength of 450 nm using a Spectra MAX 190 microplate reader (Molecular Devices, Sunnyvale, CA, USA). There were eight replicate wells for each treatment group, and this experiment was conducted in triplicate.

### 2.4. Cell Proliferation Analysis

The cell proliferation was detected using the BeyoClick^TM^ EdU Cell Proliferation Kit with Alexa Fluor 594 (Beyotime, Shanghai, China). In this experiment, cells were placed into a 96-well microplate at a concentration of 1 × 10^4^ cells/well and kept in complete medium at 37 °C and 5% CO_2_ until they reached an approximate confluence of 80%. Then, cells were treated either with or without 4.0 μM SeMet, in combination with or without simultaneous treatment with 0.5 μg/mL DON, for a duration of 24 h. Then, the cells were stained in accordance with the manufacturer’s instructions, and photographs of cells were captured utilizing a fluorescence-inverted microscope (Olympus IX73, Olympus, Tokyo, Japan), specifically in the blue fluorescence and red fluorescence channels. The red fluorescence signifies the nucleus of proliferating cells (EdU-positive cells), while the blue fluorescence indicates the entirety of the cell nucleus (Hoechst 33342-positive cells). The Hoechst 33342-positive and EdU-positive cells were quantified using the ImageJ software (1.53t version, Bethesda, NIH, Framingham, MA, USA). The cell proliferation rate was expressed as the ratio of EdU-positive cells to Hoechst 33342-positive cells (total cells). There were five replicate wells for each treatment group, and three fields were captured for each replicate well. This experiment was performed in duplicate.

### 2.5. Lactate Dehydrogenase (LDH) Release Analysis

The LDH release was detected using a commercial LDH assay kit (Beyotime, Shanghai, China). In this study, the cells were cultured and subjected to identical experimental treatments as the analysis of cell proliferation. Afterward, the 96-well microplate was centrifugated at a speed of 400× *g* for 5 min, and the supernatant was carefully removed and transferred into a new 96-well microplate. Thereafter, 60 μL of the LDH working solution was added to the microplate and incubated for 30 min at room temperature, while ensuring it remained shielded from light. Ultimately, the absorbance was assessed at a wavelength of 490 nm using a Spectra MAX 190 microplate reader (Molecular Devices, Sunnyvale, CA, USA). There were eight replicate wells for each treatment group, and this experiment was conducted in duplicate.

### 2.6. Intracellular Reactive Oxygen Species (ROS) Analysis

The intracellular ROS level was determined using a ROS assay kit (Beyotime, Shanghai, China). The ROS level is detected using a fluorescent probe (DCFH-DA). DCFH-DA does not possess any fluorescence and can traverse the cell membrane. Once inside the cell, it undergoes a hydrolyzation by esterases to form DCFH. Unlike DCFH-DA, DCFH is incapable of crossing the cell membrane in order to facilitate the loading of the probe into the cell. The presence of intracellular ROS causes the conversion of non-fluorescent DCFH into fluorescent DCF. Hence, the measurement of DCF fluorescence serves as an indicator of the cellular ROS level. In this experiment, the cells were cultured and subjected to identical experimental treatments in the analysis of cell proliferation. After the experimental treatments, a quantity of 50 μL diluted DCFH-DA (10 μM) was added to every well of the 96-well microplate and kept for a duration of 20 min at a temperature of 37 °C. Then, the 96-well plate was placed in Olympus IX73 laser confocal microscope (Olympus, Tokyo, Japan) to facilitate observation and photograph capture. The fluorescence values were calculated using the ImageJ 1.53t software. There were five replicate wells for each treatment group, and this experiment was conducted in duplicate.

### 2.7. RNA Isolation and RT-qPCR Analysis

The IPEC-J2 cells were seeded into 6-well microplates at a concentration of 4 × 10^5^ cells/well and incubated in the complete medium until they achieved an approximate confluence level of 80%. Following this, the cells were subjected to a treatment involving either the presence or absence of 4.0 μM SeMet, along with or without a concurrent treatment of 0.5 μg/mL DON, for a duration of 24 h. Then, the total RNA of cells was isolated, utilizing the TransZol Up Plus RNA Kit (TransGen Biotech, Beijing, China). After the total RNA was qualified by measuring the quality and concentration, the cDNA was synthesized using the *TransScript^®^* Uni All-in-One First-Strand cDNA Kit (TransGen Biotech, Beijing, China). Subsequently, RT-qPCR was performed on a QuanStudio^TM^ 5 Real-Time PCR Instrument (Thermo Fisher Scientific, MA, USA) using the *PerfectStart^®^* Uni RT & qPCR kit (TransGen Biotech, Beijing, China). The primers utilized for RT-qPCR were designed using the Prime Premier 5.0 software (Premier, Toronto, ON, Canada), based on the pig gene sequences in GeneBank. The synthesis of these primers was carried out by Shanghai Generay Biotech Co., Ltd. (Shanghai, China). The primer sequences are listed in Table 1. The amplification efficiency of the primers was determined by using the formula E = 10^(−1/slope of the line)−1^, as described by Pfaffl et al. (2001) [27], and the amplification efficiency of the primers was between 90% and 110%. The mRNA relative expression of the selected genes was normalized to the *β-actin* expression and calculated using the 2^−ΔΔCt^ method.

### 2.8. Western Blotting Analysis

The cells were cultured and subjected to the same experimental treatments as in the RT-qPCR analysis. Then, the total protein was extracted by the RIPA buffer containing the protease and phosphatase inhibitor cocktail. The protease and phosphatase inhibitors were purchased from Solarbio Technology Company (Solarbio, Beijing, China). The protein concentration of the extracted samples was quantified using the BCA protein assay kit (Beyotime, Shanghai, China). Before loading, each protein sample was mixed with the protein loading buffer and heated at 95 °C for 5 min. Next, the protein samples were loaded onto the gel for Sodium Dodecyl Sulfate-Polyacrylamide Gel Electrophoresis (SDS-PAGE), ensuring an equal volume and amount. Subsequently, the proteins were transferred to the Methanol-activated Polyvinylidene Fluoride (PVDF) membrane. The PVDF membranes were blocked using a 5% skim milk powder for 1.5 h at room temperature. Afterward, they were incubated with the respective primary antibodies for Erk1/2, p-Erk1/2, JNK, p-JNK, p38, p-p38, and β-actin at room temperature for 2 h. Finally, appropriate secondary antibodies were added and incubated for 2 h at room temperature. The information on primary antibodies utilized for Western blotting is listed in Table 2. *β*-actin was used as an internal reference protein. Finally, the signal was detected using an enhanced chemiluminescence system and quantified using the ImageJ 1.53t software.

### 2.9. Statistical Analysis

The statistical analysis was carried out using SPSS 25.0 software (IBM, Chicago, IL, USA). A one-way ANOVA analysis was performed, followed by Duncan’s multiple range test for making multiple comparisons. A significance level of *p* < 0.05 was used as the criteria to detect significant differences.

## 3. Results

### 3.1. The Cell Viability of IPEC-J2 Cells in Response to Toxic Dosages of DON and Physiological (or Supra-Physiological) Dosages of Selenium as SeMet

**Figure 1:**
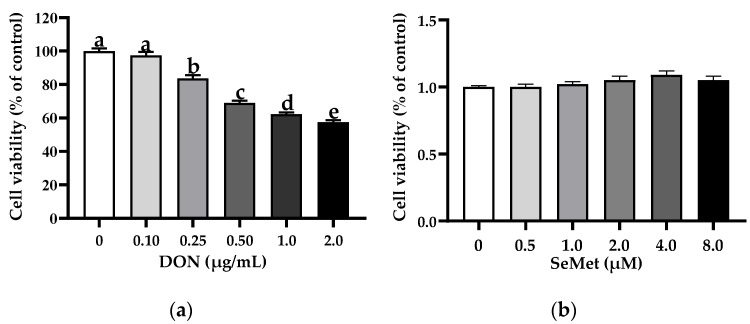
Effect of DON and SeMet on cell viability of IPEC-J2 cells. (**a**) Effect of DON on cell viability of IPEC-J2 cells. (**b**) Effect of SeMet on cell viability of IPEC-J2 cells. Cells were treated with different concentrations of DON (0, 0.1, 0.25, 0.50, 1.0, or 2.0 μg/mL) or SeMet (0, 0.5, 1.0, 2.0, 4.0, or 8.0 μM) for 24 h, and then cell viability was assessed by utilizing the CCK-8 assay. The obtained results are expressed as mean ± SEM. There were eight replicate wells for each treatment group, and this experiment was conducted in triplicate. Distinguishing letters in superscript denote a statistically significant distinction (*p* < 0.05).

The effect of DON on the cell viability of IPEC-J2 cells is shown in Figure 1a. In comparison to the control group (0 μg/mL DON group), the treatments of DON exhibited a notable inhibition on the cell viability of IPEC-J2 cells in a concentration-dependent manner. Specifically, the cell viability of IPEC-J2 cells experienced a significant decrease as the concentration of DON increased. Interestingly, the percentage of cell viability dropped below 70% when the DON concentration exceeded 0.50 μg/mL. As such, a dosage of 0.50 μg/mL was chosen for the DON treatment for the subsequent experiments. As shown in Figure 1b, in comparison to the control group (0 μM SeMet group), the treatments of different concentrations of SeMet did not affect the cell viability of IPEC-J2 cells (*p* > 0.05).

### 3.2. Effect of SeMet on Cell Viability of IPEC-J2 Cells under DON Exposure

**Figure 2:**
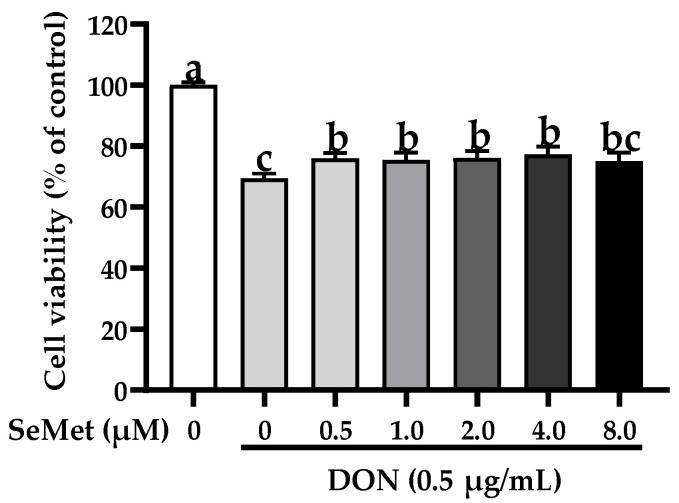
Effect of SeMet on cell viability of IPEC-J2 cells exposed to DON. Cells were cotreated with DON (0.50 μg/mL) and different concentrations of SeMet (0, 0.50, 1.0, 2.0, 4.0, or 8.0 μg/mL) for 24 h, and then cell viability was assessed by utilizing the CCK-8 assay. The obtained results are expressed as mean ± SEM. There were eight replicate wells for each treatment group, and three fields were captured for each replicate well. This experiment was conducted in triplicate. Distinguishing letters in superscript denote a statistically significant distinction (*p* < 0.05).

The effect of SeMet on the cell viability of IPEC-J2 cells exposed to DON is displayed in Figure 2. Compared with the control group, the exposure to DON markedly reduced the cell viability of IPEC-J2 cells (*p* < 0.05). Nonetheless, under the DON exposure, the cell viability was statistically elevated by 9.46%, 8.75%, 9.69%, and 11.42% when cells were treated with 0.5, 1.0, 2.0, and 4.0 μM SeMet in comparison to the group receiving only DON treatment (*p* < 0.05). Interestingly, the cell viability was not increased when treated with 8.0 μM SeMet in comparison to the DON group (*p* > 0.05). As a result, 4.0 μM was chosen as the supplementary dosage of SeMet for the subsequent experiment.

### 3.3. Effect of SeMet on Cell Proliferation of IPEC-J2 Cells under DON Exposure

**Figure 3:**
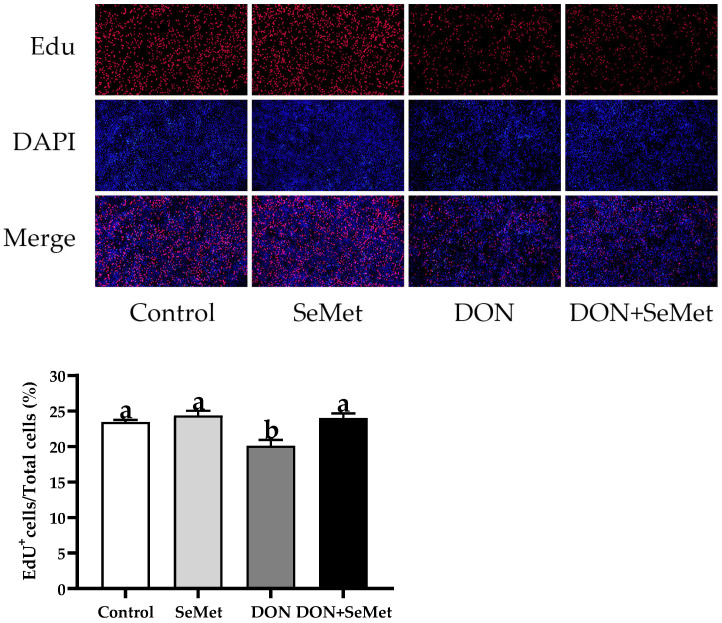
Effect of SeMet on cell proliferation of IPEC-J2 cells exposed to DON. Cells were treated either with or without 4.0 μM SeMet, in combination with or without simultaneous treatment with 0.5 μg/mL DON, for a duration of 24 h. Subsequently, cell proliferation was evaluated by employing an EdU cell proliferation kit. The red fluorescence signifies the nucleus of proliferating cells, while the blue fluorescence indicates the entirety of the cell nucleus. The cell proliferation rate is expressed as the proportion of red fluorescent nuclei to blue fluorescent nuclei. The obtained results are expressed as mean ± SEM. There were five replicate wells for each treatment group, and three fields were captured for each replicate well. This experiment was conducted in duplicate. Distinguishing letters in superscript denote a statistically significant distinction (*p* < 0.05).

As displayed in Figure 3, the cell proliferation of IPEC-J2 cells was not affected by the SeMet treatment in the absence of the exposure to DON (*p* > 0.05). The presence of DON caused a considerable reduction in the cell proliferation of IPEC-J2 cells, in comparison to the control group (*p* < 0.05). Nevertheless, when exposed to DON, the concurrent administration of SeMet statistically enhanced the cell proliferation by 19.42% in IPEC-J2 cells, in comparison to the DON group (*p* < 0.05).

### 3.4. Effect of SeMet on LDH Release of IPEC-J2 Cells under DON Exposure

**Figure 4:**
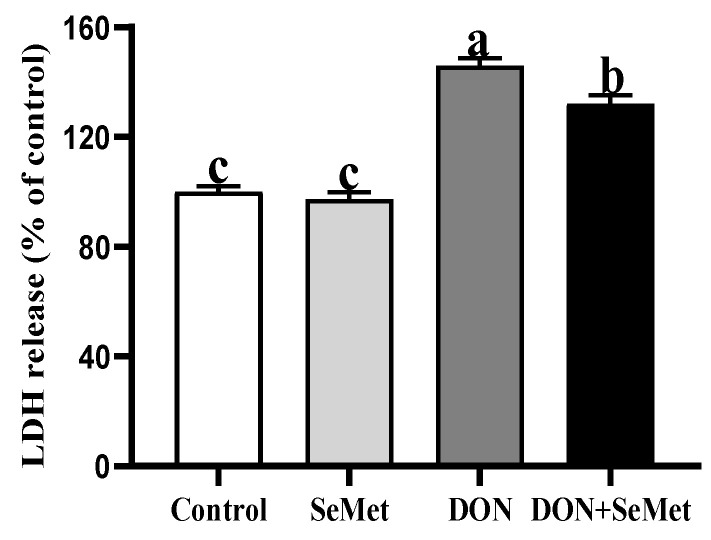
Effect of SeMet on LDH release of IPEC-J2 cells exposed to DON. Cells were treated either with or without 4.0 μM SeMet, in combination with or without simultaneous treatment with 0.5 μg/mL DON, for a duration of 24 h. Subsequently, LDH release was evaluated by employing an LDH analysis kit. The obtained results are expressed as mean ± SEM. There were eight replicate wells for each treatment group, and this experiment was conducted in duplicate. Distinguishing letters in superscript denote a statistically significant distinction (*p* < 0.05).

As shown in Figure 4, in the absence of exposure to DON, SeMet was found to have no significant impact on the LDH release by IPEC-J2 cells (*p* > 0.05). The exposure of DON resulted in a significant increase in LDH release by IPEC-J2 cells when compared to the control group (*p* < 0.05). However, in the presence of DON exposure, the cotreatment of SeMet statistically reduced the LDH release by 9.47% in IPEC-J2 cells, as compared to the DON group (*p* < 0.05).

### 3.5. Effect of SeMet on ROS Level in IPEC-J2 Cells under DON Exposure

**Figure 5:**
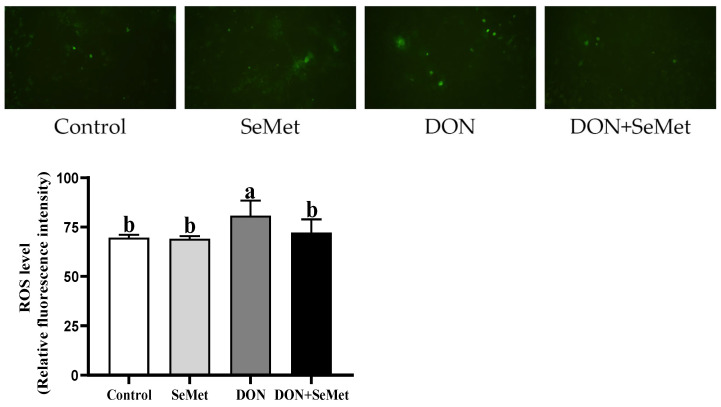
Effect of SeMet on intracellular ROS production in IPEC-J2 cells exposed to DON. Cells were treated either with or without 4.0 μM SeMet, in combination with or without simultaneous treatment with 0.5 μg/mL DON, for a duration of 24 h. Subsequently, the ROS level was evaluated by employing a ROS analysis kit. The obtained results are expressed as mean ± SEM. There were five replicate wells for each treatment group, and this experiment was conducted in duplicate. Distinguishing letters in superscript denote a statistically significant distinction (*p* < 0.05).

As depicted in Figure 5, the level of ROS in IPEC-J2 cells was unaffected by the SeMet treatment in the absence of the exposure to DON (*p* > 0.05). The presence of DON resulted in a significant increase in the ROS level of IPEC-J2 cells compared to the control group (*p* < 0.05). Interestingly, the administration of SeMet concurrently with the DON exposure reduced the ROS level by 10.57% in IPEC-J2 cells compared to the DON group (*p* < 0.05).

### 3.6. Effect of SeMet on the mRNA Relative Expression of Four Selenoproteins in IPEC-J2 Cells under DON Exposure

**Figure 6:**
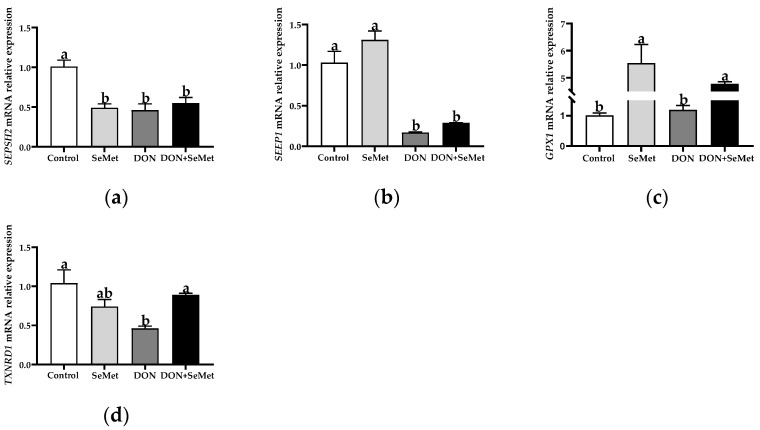
Effect of SeMet on mRNA relative expression of four selenoproteins in IPEC-J2 cells exposed to DON. (**a**) *SEPSH2* relative mRNA level. (**b**) *SEPP1* relative mRNA level. (**c**) *GPX1* relative mRNA level. (**d**) *TXNRD1* relative mRNA level. Cells were treated either with or without 4.0 μM SeMet, in combination with or without simultaneous treatment with 0.5 μg/mL DON, for a duration of 24 h. Subsequently, cells were collected for mRNA relative expression analysis of genes using RT-qPCR. All data are presented as mean ± SEM. There were six replicates for each treatment group. The bars with different letters indicate significant differences among treatments.

Compared with the control group, the SeMet treatment resulted in a significant down-regulation in the *SEPSH2* mRNA relative expression, but an up-regulation in the *GPX1* mRNA relative expression in IPEC-J2 cells (*p* < 0.05) (Figure 6). Furthermore, the exposure to DON led to a significant down-regulation in the mRNA relative expression of *SEPSH2*, *SEPP1*, and *TXNRD1* in IPEC-J2 cells, in comparison to the control group (*p* < 0.05). However, in contrast to the DON group, the cotreatment of SeMet and DON significantly up-regulated the mRNA relative expression of *GPX1* and *TXNRD1* in IPEC-J2 cells (*p* < 0.05).

### 3.7. Effect of SeMet on the mRNA Relative Expression of Antioxidant-Related Genes in IPEC-J2 Cells under DON Exposure

**Figure 7:**
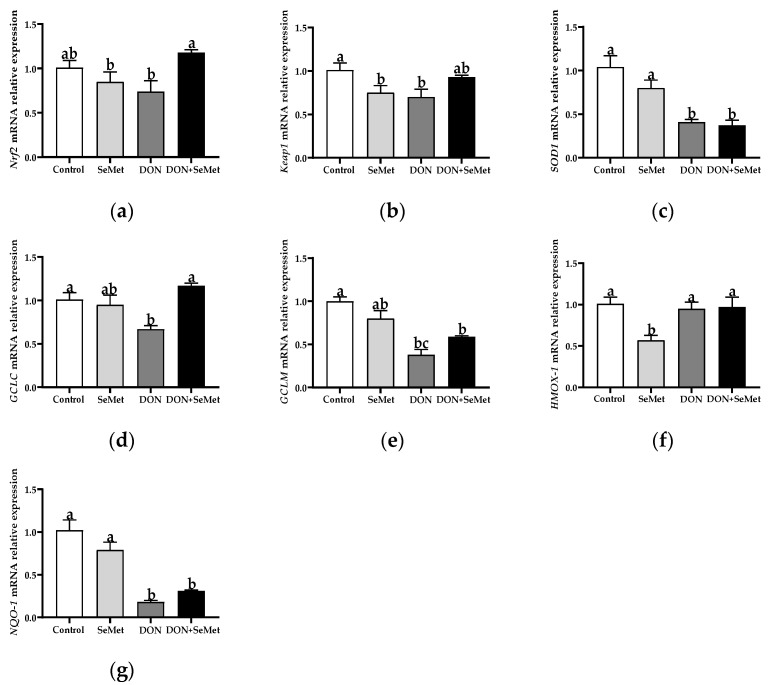
Effect of SeMet on mRNA relative expression of antioxidant-related genes in IPEC-J2 cells exposed to DON. (**a**) *Nrf2* relative mRNA level. (**b**) *Keap1* relative mRNA level. (**c**) *SOD1* relative mRNA level. (**d**) *GCLC* relative mRNA level. (**e**) *GCLM* relative mRNA level. (**f**) *HMOX-1* relative mRNA level. (**g**) *NQO-1* relative mRNA level. Cells were treated either with or without 4.0 μM SeMet, in combination with or without simultaneous treatment with 0.5 μg/mL DON, for a duration of 24 h. Subsequently, cells were collected for mRNA relative expression analysis of genes using RT-qPCR. All data are presented as mean ± SEM. There were six replicates for each treatment group. The bars with different letters indicate significant differences among treatments.

As shown in Figure 7, in comparison with the control group, the SeMet treatment markedly down-regulated the mRNA relative expression of *Keap1* and *HMOX-1* in IPEC-J2 cells (*p* < 0.05). In contrast to the control group, the DON exposure significantly down-regulated the mRNA relative expression of *Keap1*, *SOD1*, *GCLC*, *GCLM*, and *NQO1* in *IPEC-J2* cells (*p* < 0.05). Nevertheless, in contradiction to the DON group, the coadministration of SeMet and DON noticeably up-regulated the mRNA relative expression of *Nrf2* and *GCLC* in IPEC-J2 cells (*p* < 0.05). Moreover, the mRNA relative expression of *HMOX-1* was unaffected by the SeMet treatment in the presence of the DON exposure, but was down-regulated by the SeMet treatment in the absence of the DON exposure (*p* < 0.05).

### 3.8. Effect of SeMet on the mRNA and Protein Expression of Tight Junction Proteins in IPEC-J2 Cells under DON Exposure

**Figure 8:**
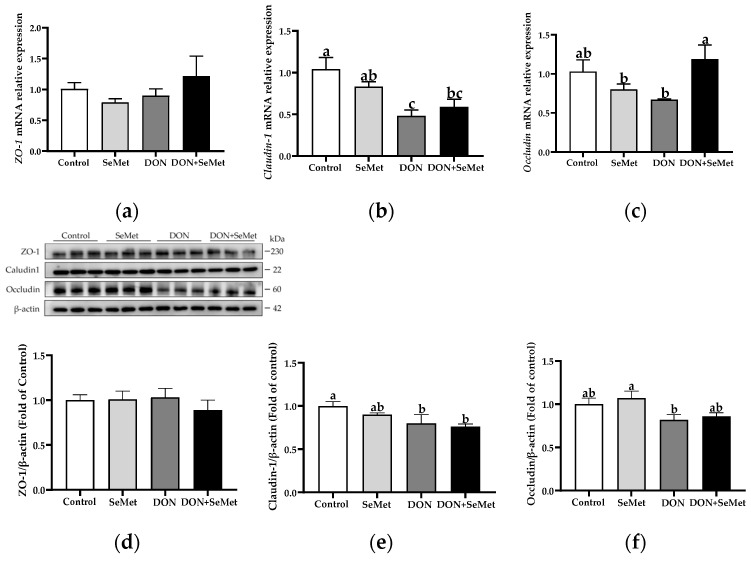
Effect of SeMet on mRNA expression of tight junction proteins in IPEC-J2 cells exposed to DON. (**a**) *ZO-1* relative mRNA level. (**b**) *Claudin-1* relative mRNA level. (**c**) *Occludin* relative mRNA level. (**d**) ZO-1 relative protein level. (**e**) Claudin-1 relative protein level. (**f**) Occludin relative protein level. Cells were treated either with or without 4.0 μM SeMet, in combination with or without simultaneous treatment with 0.5 μg/mL DON, for a duration of 24 h. Subsequently, cells were collected for mRNA relative expression of genes using RT-qPCR, or collected for protein expression using Western blotting. All data are presented as mean ± SEM. There were six replicates for each treatment group. The bars with different letters indicate significant differences among treatments.

As presented in Figure 8a–c, in the absence of exposure to DON, SeMet had no significant effect on the mRNA relative expression of *ZO-1*, *Claudin-1*, and *Occludin* in IPEC-J2 cells (*p* > 0.05). However, when cells were exposed to DON, there was a significant down-regulation in the *Claudin-1* mRNA expression compared to the control group (*p* < 0.05). Interestingly, in the presence of the DON exposure, the concurrent treatment with SeMet significantly upregulated the *Occludin* mRNA relative expression in IPEC-J2 cells (*p* < 0.05). As shown in Figure 8d,e, when compared to the control group, the DON exposure significantly down-regulated the Claudin-1 protein expression in IPEC-J2 cells (*p* < 0.05). However, in both the absence and presence of exposure to DON, the treatment with SeMet did not affect the protein expression of ZO-1, Claudin-1, and Occludin in IPEC-J2 cells (*p* > 0.05).

### 3.9. Effect of SeMet on Protein Expression of Key Proteins in MAPK Pathway in IPEC-J2 Cells under DON Exposure

**Figure 9:**
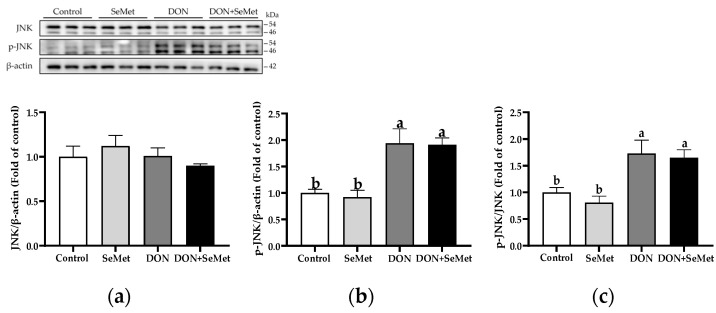
Effect of SeMet on protein expression of JNK and p-JNK in IPEC-J2 cells exposed to DON. (**a**) JNK relative protein level. (**b**) p-JNK relative protein level. (**c**) The ratio of p-JNK to JNK protein level. Cells were treated either with or without 4.0 μM SeMet, in combination with or without simultaneous treatment with 0.5 μg/mL DON, for a duration of 24 h. Subsequently, cells were collected for protein expression of JNK and p-JNK using Western blotting. All data are presented as mean ± SEM. There were six replicates for each treatment group. The bars with different letters indicate significant differences among treatments.

In both the absence and presence of exposure to DON, SeMet treatment did not affect the protein expression of JNK and phosphorylated JNK in IPEC-J2 cells (*p* > 0.05) (Figure 9). Nonetheless, when cells were subjected to DON, there was a significant up-regulation in the phosphorylated JNK protein level and the ratio of phosphorylated JNK to JNK protein level compared to the control group in IPEC-J2 cells (*p* < 0.05).

**Figure 10:**
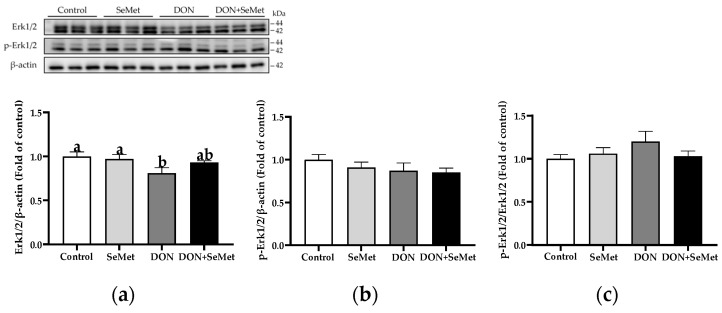
Effect of SeMet on protein expression of Erk1/2 and p-Erk1/2 in IPEC-J2 cells exposed to DON. (**a**) Erk1/2 relative protein level. (**b**) p-Erk1/2 relative protein level. (**c**) The ratio of p-Erk1/2 to Erk1/2 protein level. Cells were treated either with or without 4.0 μM SeMet, in combination with or without simultaneous treatment with 0.5 μg/mL DON, for a duration of 24 h. Subsequently, cells were collected for protein expression of Erk1/2 and p-Erk1/2 using Western blotting. All data are presented as mean ± SEM. There were six replicates for each treatment group. The bars with different letters indicate significant differences among treatments.

In the absence as well as the presence of exposure to DON, treatment with SeMet had no impact on the protein expression of Erk1/2, phosphorylated Erk1/2, and the ratio of phosphorylated Erk1/2 to Erk1/2 protein level in IPEC-J2 cells (*p* > 0.05) (Figure 10). However, when cells were exposed to DON, there was a reduction in the protein expression of Erk1/2 as compared to the control group (*p* < 0.05).

**Figure 11:**
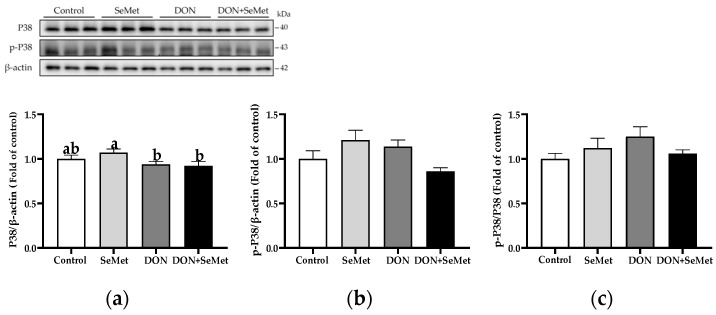
Effect of SeMet on protein expression of P38 and p-P38 in IPEC-J2 cells exposed to DON. (**a**) P38 relative protein level. (**b**) p-P38 relative protein level. (**c**) The ratio of p-P38 to P38 protein level. Cells were treated either with or without 4.0 μM SeMet, in combination with or without simultaneous treatment with 0.5 μg/mL DON, for a duration of 24 h. Subsequently, cells were collected for protein expression of P38 and p-P38 using Western blotting. All data are presented as mean ± SEM. There were six replicates for each treatment group. The bars with different letters indicate significant differences among treatments.

In both the absence and presence of the exposure to DON, treatment with SeMet did not have a significant effect on the protein expression of P38, phosphorylated P38, and the ratio of the phosphorylated P38 to P38 protein level in IPEC-J2 cells (*p* > 0.05) (Figure 11). Moreover, when compared to the control group, the DON exposure did not statistically alter the protein expression of P38, phosphorylated P38, and the ratio of phosphorylated P38 to P38 protein level in IPEC-J2 cells (*p* > 0.05).

## 4. Discussion

The DON toxin is produced by the *Fusarium* fungi and categorized within the trichothecene B family of toxins, which also includes nivalenol, trichothecin, and fusarenon X [28]. DON is a prevalent mycotoxin that contaminates grains, animal feed, and food products. After the consumption of diets contaminated with DON, the animals’ intestinal tract will be exposed to an environment with a substantial concentration of DON. It has been demonstrated that the gastrointestinal epithelial barrier plays a crucial role in the prevention of food contamination and entry of intestinal pathogens. The impairment of the intestinal barrier will result in the occurrence of intestinal diseases and increase in pathogen infiltration [29]. According to previous studies, the presence of DON has been found to potentially provoke oxidative stress, disrupt epithelial tight junctions, and trigger inflammation and apoptosis, as well as induce intestinal barrier dysfunction [30,31,32]. In the present study, the treatments of DON significantly hindered the cell viability of IPEC-J2 cells in a dose-dependent manner, which aligns with previous studies [33,34,35]. It is worth noting that the cell viability percentage plummeted to less than 70% when the concentration of DON went above 0.50 μg/mL. Therefore, a dosage of 0.50 μg/mL was selected for DON treatment in the subsequent experiments.

Selenium plays a vital role in the antioxidant defense system, and it is an indispensable micronutrient for both humans and animals [36,37]. SeMet is an organic derivative of selenium and serves as an excellent source of dietary selenium for swine nutrition. It has been reported that when compared to the administration of 0.3 mg/kg selenium as sodium selenite, the addition of 0.3 mg/kg selenium as Se-enriched yeast enhanced the reproductive performance and antioxidant status of sows [13]. Furthermore, another study found that the dietary supplementation of 0.3 mg/kg selenium as SeMet in late gestation and lactation increased the selenium content and antioxidant status in the milk of sows [38]. More interestingly, SeMet has been found to effectively counteract the oxidative damage and cell death observed in IPEC-J2 cells that was caused by an exposure to zearalenone [19]. In the current study, it was observed that under the exposure of DON, the cell viability of IPEC-J2 cells significantly increased when cells were subjected to the treatment of 0.5, 1.0, 2.0, and 4.0 μM SeMet, as compared to the group that received only a DON treatment. It should be noted that the cell viability was not increased when treated with 8.0 μM of SeMet in comparison to the DON group, which may suggest that 8.0 μM selenium is a supraphysiological dosage for pigs. Further research is warranted to assess the potential toxicity of supra-physiological or super-nutritional levels of SeMet in pigs. Henceforth, 4.0 μM has been selected as the SeMet dosage for the subsequent experiment.

To further explore the protective effects of SeMet on DON-induced intestinal cytotoxicity, cell proliferation and LDH release were evaluated in IPEC-J2 cells under DON exposure. LDH release is an indicator of cell toxicity [39]. A commonly utilized approach in determining cytotoxicity involves measuring the activity of cytoplasmic enzymes that are released as a result of cell damage [39]. LDH is a constant cytoplasmic enzyme present in all cells. The LDH release into the cell culture supernatant occurs swiftly upon the occurrence of damage to the plasma membrane, representing a vital characteristic observed in cells experiencing cellular injury [39]. In the current experiment, it has been observed that DON significantly enhanced the cellular LDH release while suppressing cell proliferation, which is in accordance with previous studies [21,24]. Interestingly, in comparison to the treatment of DON alone, the co-treatment of SeMet and DON exhibited a reduction in LDH release, thereby mitigating the inhibitory impact of DON on cell proliferation. The above-observed results indicate that SeMet exerts an important protective role in DON-induced cytotoxicity regarding promoting cell proliferation and decreasing cytotoxicity in IPEC-J2 cells.

The ROS is generated by numerous physiological processes within the cells. The ROS level in normal cells remains in a state of dynamic equilibrium due to the influence exerted by pro-oxidation and anti-oxidation systems [40]. When cells are stimulated by external stimuli, the level of ROS elevates, surpassing the antioxidant capacity. Subsequently, the cells enter a state characterized by oxidative stress, wherein mitochondrial damage, metabolic disorders, and even apoptosis may occur [41]. Previous studies have shown that DON can increase intracellular ROS levels and up-regulate the expression of genes and proteins related to apoptosis and inflammation [33,42,43]. In our study, the presence of DON resulted in a significant increase in the ROS level of IPEC-J2 cells compared to the control group. However, the administration of SeMet concurrently with the DON exposure led to a significant decrease in the ROS level in IPEC-J2 cells compared to the DON group. The abovementioned findings suggest that SeMet possesses the ability to significantly reduce the excessive production of ROS and alleviate the oxidative stress caused by the exposure to DON in IPEC-J2 cells.

Selenoproteins are a family of proteins that encompass the amino acid selenocysteine, which performs a crucial function in human and animal health [44]. A plethora of selenoproteins are engaged in redox processes, which serve as fundamental enzymatic antioxidant systems [45,46]. It was previously demonstrated that the first three classes of eukaryotic selenoproteins discovered are glutathione peroxidases, thioredoxin reductases, and deiodinases [47]. Moreover, it was demonstrated that the GPXs serve as the primary selenoenzymes and constitute an essential element of antioxidant glutathione pathways, offering a defense against ROS. The GPX1 emerged as one of the most abundant and ubiquitously expressed selenoproteins [48]. The GPX1, also expressed in IPEC-J2 cells, is an antioxidant selenoprotein that exhibits a remarkable sensitivity to alterations in selenium levels, along with conditions of oxidative stress [49]. This antioxidant enzyme employs glutathione as a substrate for the detoxification process of hydrogen peroxide, which plays a pivotal role in regulating cellular activities influenced by hydroperoxides [49]. The SEPSH2 is the key regulatory protein in selenoproteins’ synthesis [48], while SEEP1 is the major transporter of selenium from the liver to other tissues, and has also been demonstrated to possess antioxidant properties [50]. Additionally, the thioredoxin reductases are catalysts of oxidation-reduction reactions, which play crucial roles in cellular redox processes. Their function involves the reduction in thioredoxin by utilizing NADPH [51,52]. In the present study, the exposure to DON led to a significant down-regulation in the mRNA relative expression of *SEPSH2*, *SEPP1*, and *TXNRD1* in IPEC-J2 cells, in comparison to the control group. Those findings indicate that the DON exposure reduced the selenoprotein synthesis. Moreover, in contrast to the DON group, the cotreatment of SeMet and DON significantly up-regulated the mRNA relative expression of *GPX1* and *TXNRD1* in IPEC-J2 cells, which suggests that SeMet promoted the two main antioxidant selenoproteins, and thus enhanced the cellular antioxidant capacity to defend against the oxidative stress induced by DON. However, it should be noted that, in the absence of DON exposure, the SeMet treatment resulted in a down-regulation of the *SEPSH2* mRNA expression in IPEC-J2 cells. This surprising result may be related to the negative feedback on selenoproteins’ synthesis, which is consistent with the results of Liu et al. (2021) [53]. The aforementioned results suggest that SeMet possesses the capability to alleviate the noxious impacts of DON by augmenting the efficacy of antioxidant selenoproteins.

The Nrf2/Keap1 signaling pathway functions as a defense mechanism to maintain cellular homeostasis; it is also a primary cellular response mechanism to the toxicity induced by mycotoxins [54,55]. It is evident that the Nrf2/Keap1 signaling pathway is capable of regulating over 100 genes downstream, with a specific emphasis on genes associated with anti-redox signaling, anti-oxidative stress, and cell protection target genes [56]. In the current study, compared with the control group, the exposure to DON markedly down-regulated the mRNA relative expression of *Keap1*, *SOD1*, *GCLC*, *GCLM*, and *NQO1* in IPEC-J2 cells. These findings align with the results, indicating that DON induces an oxidative stress injury, as demonstrated by the elevated levels of ROS and increased release of LDH in the DON group of IPEC-J2 cells. Moreover, the mRNA relative expression of *HMOX-1* was unaffected by SeMet treatment in the presence of the DON exposure, but was down-regulated by the SeMet treatment in the absence of the DON exposure. These results could explain that the *HMOX-1* expression response by selenium supplementation may be related to different stimuli stress conditions. For instance, Miyata et al. (2021) found that the hepatic mRNA expression abundance of *HMOX-1* was down-regulated (0.19 vs. 1.00) by a selenoneine supplementation in mice with non-alcoholic fatty liver disease [57]. Eddie-Amadi et al. (2023) also noted that the sodium selenite treatment decreased the HMOX-1 level in the thyroid of rats under a heavy metal mixture exposure [58]. However, Al-Brakatiet al. (2021) reported that the sodium selenite or lycopene-coated selenium nanoparticles up-regulated the mRNA expression abundance of *HMOX-1* in the kidney of rats in a glycerol-induced acute kidney injury model [59]. Nevertheless, in contradiction to the DON group, the coadministration of SeMet and DON noticeably up-regulated the mRNA relative expression of *Nrf2* and *GCLC* in IPEC-J2 cells. Consistent with our results, Ju et al. (2021) found that SeMet activated the Nrf2 pathway in the duodenum and jejunum of broilers, and significantly up-regulated the mRNA levels of antioxidant enzymes downstream of the Nrf2 pathway, thus effectively mitigating the fluorine-induced apoptosis in the intestine of broilers [60]. Similarly, Sun et al. (2021) also demonstrated that SeMet mitigated the oxidative stress injury provoked by zearalenone through the activation of the Nrf2/Keap1 pathway in IPEC-J2 cells [19]. The up-regulation of the *Occludin* mRNA level, which indicates the function of the intestinal barrier, also demonstrates the antioxidant protection provided by SeMet [61,62].

It has been reported that the exposure to DON induced the selective activation of mitogen-activated protein kinase (MAPK) pathways [22]. Consequently, a Western blotting analysis was performed to determine the expression levels of pivotal proteins in MAPK pathways in IPEC-J2 cells under a DON exposure. The MAPKs, including the p38, Erk1/2, and JNK cascades, are key pathways that regulate a wide variety of cellular processes [63]. Zhang et al. (2020) found that the DON exposure resulted in cellular inflammation in IPEC-J2 cells through the activation of P38 Mapk and Erk1/2 pathways [34]. Yu et al. (2021) also documented that the DON treatment triggered the inflammation response in IPEC-J2 cells through the phosphorylation of MAPK signaling pathways [63]. Consistently, the present study demonstrates that DON is capable of promoting the phosphorylation of JNK protein expression, which indicates that DON activates the JNK MAPK pathway in IPEC-J2 cells. However, in the present study, in both the absence and presence of exposure to DON, the SeMet treatment did not affect the protein expression of MAPK (JNK, Erk1/2, and P38), phosphorylated MAPK (p-JNK, p-Erk1/2, and p-P38) in IPEC-J2 cells. This indicates that SeMet alleviated the DON-induced oxidative injury in porcine intestinal epithelial cells regardless of the MAPK pathway regulation. Lastly, it is important to acknowledge the limitations of the current study. Although the protective effects of SeMet on the DON-induced cell toxicity are statistically significant, the inhibitory effects of SeMet on DON are observed to be approximately between 10% and 20% (including cell viability, cell proliferation, LDH release, and ROS level). Hopefully, this research provides a reference for the intestinal oxidative stress induced by DON, or other potential sources of oxidative stress, such as heat stress or other mycotoxin toxicities.

## 5. Conclusions

Collectively, SeMet alleviated the DON-induced cellular toxicity in IPEC-J2 cells, including cell viability, cell proliferation, and LDH release. Furthermore, SeMet reduced the DON-caused oxidative stress, and enhanced the antioxidant capacity and antioxidant selenoprotein expression in IPEC-J2 cells. However, the protective effects of SeMet on IPEC-J2 cells is independent of the MAPK pathway regulation.

## Figures and Tables

**Table 1:** The primer sequences used for RT-qPCR.

Genes	Primer Sequence (5′-3′)	Accession Number	Product Size (bp)
*TXNRD1*	F: CAAATCGAAGCAGGGATGC R: TCGTCACTGTTAGTGGCCTT	NM_214154.3	62
*SEPHS2*	F: CTGAGTGACCTCTACGCCAT R: GCATCCCGAAAGCCTTTGAT	NM_001093735.1	131
*SEPP1*	F: AGTCACCTGACAGTGTGGAG R: AATGACGTTCTCCTCTGCCA	EF113596.2	76
*Nrf2*	F: CCCATTCACAAAAGACAAACATTC R: GCTTTTGCCCTTAGCTCATCTC	XM_021075133.1	72
*Keap1*	F: GACGTGGAGACAGAAACGTG R: GTGACCATCATAGCCTCCGA	NM_001114671.1	114
*SOD1*	F: AAGGCCGTGTGTGTGCTGAA R: GATCACCTTCAGCCAGTCCTTT	NM_001190422.1	118
*GCLC*	F: GACGACGCCAATGAGTCTGA R: AGCACCACAAACACCACGTA	XM_021098556.1	173
*GCLM*	F: GATGCCGCCCGATTTAACTG R: ACAATGACCGAGTACCGCAG	XM_001926378.4	177
*HMOX-1*	F: TCAAGCAGAGGGTCCTCGAA R: CCTCTTGCGGATGTCGGATG	NM_001004027.1	134
*NQO-1*	F: GCCCAGATATTGTGGCCGAA R: AACTCCCCTATGAGCACACG	NM_001159613.1	130
*GPX1*	F: TGAATGGCGCAAATGCTCAC R: ATTGCGACACACTGGAGACC	NM_214201.1	125
*ZO-1*	F: AAGATCCGATAGGCGGTCTG R: CGGGATTTCACCAGTGTGAC	XM_021098827.1	72
*Claudin-1*	F: GGCAGATCCAGTGCAAAGTC R: CCCAGCAGGATGCCAATTAC	NM_001233539.1	94
*Occludin*	F: CATTATGCACCCAGCAACGA R: GCACATCACGATAACGAGCA	XM_005672522.3	168
*β-actin*	F: TCTGGCACCACACCTTCT R: TGATCTGGGTCATCTTCTCAC	XM_021086047.1	114

Abbreviations: *TXNRD1*, Thioredoxin reductase 1; *SEPHS2*, Selenophosphate synthetase 2; *SEPP1*, Selenoprotein P; *Nrf2*, Nuclear-factor-erythroid-2-related factor 2; *Keap1*, Kelch-like ECH-associated protein 1; *SOD1*, Superoxide dismutase 1; *GCLC*, Glutamate-cysteine ligase catalytic subunit; *GCLM*, Glutamate-cysteine ligase modifier subunit; *HMOX-1*, Heme oxygenase 1; *NQO-1*, NAD(P)H quinone dehydrogenase 1; *GPX1*, Glutathione peroxidase 1; *ZO-1*, Zonula occludens-1; *β-actin*, Beta-actin.

**Table 2:** The information of primary antibodies utilized for Western blotting.

Primary Antibody	Origin	Diluted Multiples	Brand
Erk1/2	Rabbit	1:1000	Cell Signaling Technology
p-Erk1/2	Rabbit	1:1000	Cell Signaling Technology
JNK	Rabbit	1:1000	Cell Signaling Technology
p-JNK	Rabbit	1:1000	Cell Signaling Technology
P38	Rabbit	1:1000	Cell Signaling Technology
p-P38	Rabbit	1:1000	Cell Signaling Technology
ZO-1	Rabbit	1:5000	Proteintech
Claudin-1	Rabbit	1:5000	Proteintech
Occludin	Rabbit	1:5000	Proteintech
β-actin	Mouse	1:5000	Proteintech

## Data Availability

Data are contained within the article.
